# Functional Outcomes in Upper Limb Replantation—A Systematic Review

**DOI:** 10.3390/jcm13051289

**Published:** 2024-02-24

**Authors:** Andrea Bueno, Endika Nevado-Sanchez, Carla Collazo, Raquel De la Fuente-Anuncibay, Jerónimo González-Bernal

**Affiliations:** 1 Las Huelgas Health Centre (Burgos), 09001 Burgos, Spain; abuenof@saludcastillayleon.es; 2 Reconstructive and Aesthetic Plastic Surgery Service, Burgos University Hospital, 09006 Burgos, Spain; enevados@saludcastillayleon.es; 3 Health Sciences Department, University of Burgos, 09006 Burgos, Spain; jejavier@ubu.es; 4 Education Department, University of Burgos, 09006 Burgos, Spain; raquelfa@ubu.es

**Keywords:** replantation, upper limb, functionality

## Abstract

Functionality after upper limb replantation is a decisive factor when considering the success of the intervention. Therefore, its evaluation is fundamental. The aim of this article was to conduct a systematic review of upper limb functions after replantation or reimplantation, seeking to identify reported functional outcomes as well as the level and mechanism of injury. To achieve this objective, a literature search was conducted in PubMed, ScienceDirect, Cochrane and Web of Science. Studies from the last 10 years which included patients with upper limb replantation and reported their functional outcomes were included. Out of 523 articles, 12 studies (*n* = 607) were finally included. DASH and CISS were the most commonly used assessments to report functional outcomes. In conclusion, functional outcomes after replantation are assessed using widely varying scales; therefore, due to this methodological variability, it is difficult to compare functional success between studies and further studies on functionality are needed to provide new data.

## 1. Introduction

Replantation is a complex procedure that has undergone a major evolution in recent years, thanks to advances in microsurgical techniques, which have become increasingly safer [1,2,3,4]. Since Malt and McKhann performed the first reattachment of an amputated limb in 1964, a number of questions have arisen as to whether or not reattachment should be performed [5].

In the case of the upper limb, reattachment is defined as the process of joining a body segment by arterial and venous anastomosis for the survival of completely or incompletely amputated segments [2,6].

Replantations can be an optimal solution to the problems of mutilation. The principle of the optimization of results is currently being applied through the selection of patients who are candidates for these procedures. Some authors argue that functional results cannot be sacrificed for aesthetics or other aspects, so a reimplanted limb without adequate mobility or sensibility is not considered acceptable [2,3,6,7,8,9,10].

The factors taken into account when performing replantation, in addition to the characteristics of the lesions themselves, such as their level and mechanism, include ischemia time, age, occupation, the feasibility of replantation (including the surgical technique, material, infrastructure and trained personnel), other concomitant lesions or pathologies [11,12], psychosocial factors, patient involvement and adherence to rehabilitation treatment [1].

Some authors [1] point out the importance of taking into account some general decision-making considerations; in this sense, the condition of the amputated part when it arrives at the hospital is important in considering whether to reimplant, but this is not the only determinant. Other factors must be weighed when deciding whether a part should be reimplanted, such as the overall clinical condition of the patient (including their suitability for long-term rehabilitation), the condition of the amputated part, the complexity of the microsurgery required, and the importance of the amputated part to the function and activity of the patients’ upper limbs.

An upper limb amputation is a major life event and can be devastating both physically and behaviorally [1,5].

Classification systems have been developed to guide the decision to reimplant, which are useful both for describing a lesion, preoperative planning, and for monitoring postoperative outcomes, making it possible to compare similar lesions and facilitating procedures [5,8,13,14]. In this sense, the indications for replantation are based on the probability of the survival of the reimplanted part; the safety of the procedure for the patient compared to the costs and the patient’s wishes, with the need for individual case studies and exceptions such as single-digit replantation; whether the patient is a child; whether the finger is the thumb; the mechanism of the ring avulsion injury; the level distal to the insertion; the special needs of the patient according to his profession; whether the patient already has a compromised hand; whether he has comorbidities that hinder his rehabilitation, etc. [5,13].

A successful reattachment of the upper limb is possible in 77–93% of cases [5,15,16].

As has been pointed out, the success of the intervention itself cannot be considered without taking functionality into account, as this is the main goal of replantation interventions. Furthermore, it must be taken into account that a loss of function results in a reduction of autonomy in daily life [17].

Functional outcomes have improved over the decades due to advances in medical equipment, surgical expertise and facilities. While, decades ago, replantation was not considered for crushing and avulsion injuries, new advances have resulted in up to 90% success rates of useful salvages in severe injuries [18].

The publication of case reports has allowed for the assessment of long-term functional outcomes, which has facilitated informed decisions about the reattachment of an amputated limb. In this sense, the decision to intervene should be based on the likelihood of saving the limb by providing it with utility and function, and not just on the survival of the limb [5,19].

No consensus has been found on the assessment of functionality in upper limb replantations. In this regard, Sebastin et al. point out the need for a comprehensive assessment that includes objective, subjective, health-related quality of life and psychological evaluations [20].

The aim of this work was to perform a systematic review of upper limb functionality after replantation, seeking to identify the functional outcomes reported, as well as the level and mechanism of injury.

## 2. Methods

The objectives, inclusion criteria and methods were first developed for this review, taking into account the PRISMA 2020 recommendations. To meet the inclusion criteria, studies had to include (1) patients (2) with upper limb replantation, (3) and report the functional outcomes of the replantation. We included studies published between 2013 and 2023, that is, the last 10 years, for which the full text was available. Abstracts, case studies, conference reports, literature reviews and meta-analyses were excluded.

A literature search was conducted in PubMed, ScienceDirect, Cochrane and the Web of Science. The search terms used were as follows: upper limb AND reimplant AND functional results. Studies were reviewed by two authors independently and disagreements were resolved by discussion and agreement with the lead author where necessary. The reference lists of the included articles were reviewed for additional studies that met the inclusion criteria.

Data extraction was performed systematically, using our own database. Population characteristics, injury level, injury mechanisms and functional outcomes were reviewed. Summary data were then generated.

## 3. Results

**Figure 1:**
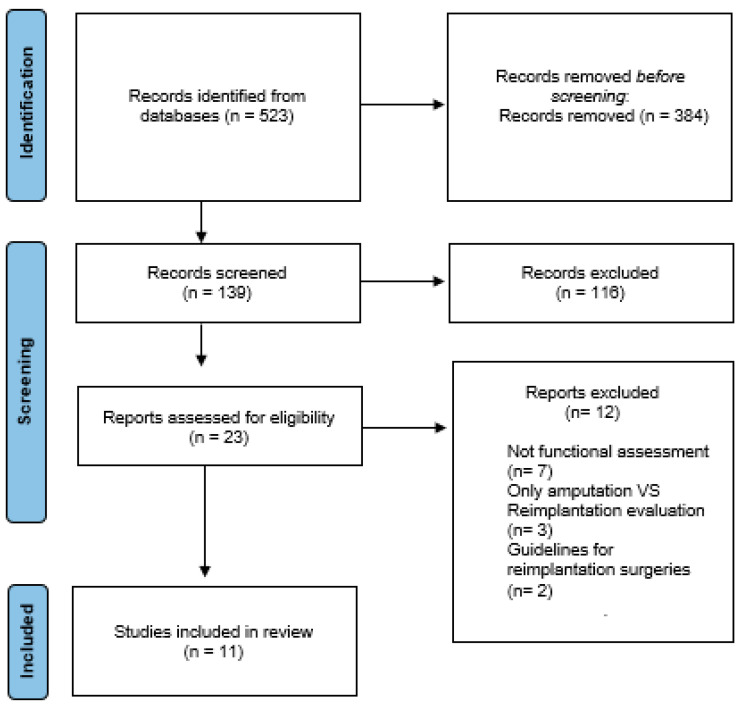
PRISMA flow diagram.

The primary search identified 523 articles. After a review of their titles and abstracts, followed by in-depth analysis of the full article, 11 articles (Table 1) were included as they met the inclusion criteria (Figure 1). A total of 607 patients who underwent replantation (84% male), with a mean age of 38 years, were included in the analysis.

The mechanism of injury was divided between a total of 407 cut injuries, 80 crush injuries, 26 avulsion injuries and 5 self-inflicted injuries, while, when talking about the level of injury, 14 arm replantations, 31 elbow and forearm, 27 wrist, 29 metacarpal, 361 digit and 144 thumb replantations were found. Demographic data can be found in Table 2.

To sum up, out of the reviewed studies, five discuss various levels of injury (as mentioned in Table 2) within the same article. Additionally, four studies focus on digit replantation, and two specifically address thumb injuries.

As for the functional outcomes reported, the main point to note is that they are highly variable. It is not possible to find a single assessment that underpins all the articles; each one administers different scales. It is true, however, that the scales that appear in most articles are the Disabilities of the Arm, Shoulder and Hand (DASH) (6/11), and the Coping Inventory for Stressful Situations (CISS) (7/11). The DASH assesses subjective distress, activity and participation restriction [21], as well as ability to perform upper limb activities [12] (activity limitations and participation restriction) [16]; the mean score found in the reviewed studies is 28.91 (6.6–75.4). Note that this scale ranges from 0 to 100, where 0 equals no disability and 100 equals severe disability [21], while the CISS [22], which assesses cold intolerance, reports 166 people affected.

The other assessment scales found are the QuickDASH [23], a short version of the DASH, which is said to be an easy and reliable way to assess functional outcomes [11], administered in three studies, reporting a mean of 24.9 (11.4–41.17); Chen’s functional criteria [24] are taken into account in two articles, where the majority of patients are found to be in grade I and II, which is a good qualification for replantation; two other studies assess functionality using the Michigan Hand Outcome Questionnaire (MHQ) [25], which assesses the subjective perception of appearance, pain and satisfaction; two others use the RAS [26] and two take into account values from the range of motion (ROM) questionnaire [27]. Only one assesses functional independence with the Functional Independence Measure (FIM) [28]; one assesses quality of life with the EQ-5D [29]; and one assesses psychological outcomes, including symptoms of depression, anxiety and post-traumatic stress disorder (Statistical Anxiety Scale (SAS) [30], Beck Depression Inventory (BDI) [31] and Post-Traumatic Stress Symptom Screening (SPTSS) [32], respectively). Table 3 lists the scales and the number of articles in which they appear.

Although, as can be seen, there is no consensus on the evaluation and presentation of the results of replantation, it can be observed in the articles evaluated, thanks to the scoring of the scales presented, that the results and rating of replantation are good and that the patients’ satisfaction, quality of life and functionality after surgery are positive.

## 4. Discussion

This study presents a review of the articles on replantation/reimplantation from 2013 to the present. A great variety is found in terms of the functional outcomes reported. That is, each study uses different scales; some use only objective scales, some use subjective scales, some take into account the psychological influence that the change in the upper limb may have, some do not. And that is why, in this sense, this review shows the need for some indications to assess the functionality of the reimplanted upper limb.

There is agreement that technical innovations increase the survival and quality of replantation or reimplantation, but it is difficult to control all the factors that affect the final functional outcome and therefore influence the decision to reimplant or not to reimplant. Different factors such as the mechanism of injury, ischemia time and other variables that have also been described, such as age, previous state of health, patient expectations, rehabilitation possibilities, social environment, etc., have been pointed out as having a strong influence on the patient’s final functional prognosis.

Different authors state that the mere survival of the reimplanted limb or fragment should not take precedence over its minimal useful function [5,6]. Although some general indications for replantation have been established [8,9,24,33], more recent studies have included new techniques such as delayed replantation [2,3,10], finding higher concordance in multiple toe, first toe, infant population and zone I long toe injuries. In the case of zone II long finger amputations, there is more controversy [7].

Regarding the relationship between the level and mechanism of injury and the limb’s functional outcome, some authors [6] point out that, in the case of reimplantation or proximal reimplantation, Chuang [8] classes I (distal to the muscle–tendon junction) and II (through muscle bellies), at the forearm level, should always be attempted (unless there is a major contraindication due to severe polytrauma, a hot ischemia time exceeding 6–8 h or a severe avulsion or crushing of the segment). Amputations at or proximal to the elbow must be assessed on an individual basis, and, in all circumstances, the joint must always be spared. With proximal amputations, there are few reconstructive possibilities, and prosthetic fitting is not always adequate (23% failure in mechanical and 26% failure in myoelectric prosthetics), to which it must be added that the rehabilitation of upper limb amputees requires very specialized units and consumes many resources [34].

However, in terms of the results provided by the studies reviewed, we found that primary amputations are better than secondary amputations [35]. Regarding functional outcomes, there are different points of view in the studies reviewed. Some authors state that it is difficult to speak to the benefits seen after reimplantation [36], as well as the important limitations and dysfunctions found after reimplantation [11,37], while others point to satisfactory reimplantation results [12,17,38,39] or the variability of their results [38].

On the other hand, different studies point out the relationship between possible indications and contraindications and functional outcomes [1,7]. Poor prognostic factors for survival and function in replantation or reimplantation include crush/victimization, ischemia time, amputation level, major bone loss, loss of peripheral nerve supply, joint involvement, advanced age, being poor candidate for immobilization, unrealistic expectations or psychosocial disturbances and poor adaptation to rehabilitation [2,3].

This review had a number of limitations. Despite following a systematic search method, it was difficult to conduct a bias analysis due to the sociodemographic variability of the samples and the assessment instruments used. This made it difficult to compare and extrapolate the articles’ results. As it is true that no other systematic reviews focusing on functional outcomes after upper limb replantation have been found, this may be the main strength of this study.

On the other hand, we have not found a consensus guide to functional outcomes, although there are different validated scales, based on objective criteria that are measurable both from an objective point of view and subjectively from the patient’s point of view. Among the most widely used are the QuicK DASH; the TMM (modified Mayo table); RMA (Range of joint mobility); the Tamai Scale; the Chen Scale, the Medical Research Council (MRC) scale; and the Russell questionnaire, among others.

It is striking that there is hardly any mention of the importance of hand therapy, in cases of reimplantation, to regain functionality. Few articles report that patients have been able to return to their pre-accident occupation/job after hand therapy [7,35]. And others point out the importance of care and rehabilitation programs to ensure a good postoperative period and reintegration into employment [7,40,41].

In general, we can differentiate the follow-up of function after replantation or replantation into different perspectives; although the objective results, in any type of amputation, of the degree of function may be low in some cases, the degree of subjective satisfaction is higher. Some authors [7,42] attribute this to the fact that reimplantation preserves bodily integrity while implementing positive emotional factors.

On the other hand, when the patient is aware of the difficulties and objectives of the procedure, they are much more involved in the prolonged rehabilitation treatment, which leads them to be more realistic and to assume an objectively low outcome.

## 5. Conclusions

Replantation/reimplantation is a complex procedure that, if well indicated, is an optimal treatment for mutilating upper extremity injuries and is preferred by patients over amputation. The optimization of outcomes requires a process of self-assessment for the patient of their functional outcomes.

The present review of the assessment of functional outcomes after reimplantation points to a wide variety of scales, which makes it difficult to compare the functional success between studies. The most severe lesions are those with the worst quality of life and worst functional outcomes.

More direct and cost-effective comparative studies are needed to determine the true impact of such interventions, not only at the individual but also at the societal level, while also addressing patients’ perceived quality of life, which will allow for more precise criteria for these interventions’ implementation.

## Figures and Tables

**Table 1:** Included articles.

Article	Type of Article	*n*
Functional Outcomes of Secondary Procedures in Upper Extremity Replantation and Revascularization	Prospective observational study	40
Long-term outcomes of major upper extremity replantations	Prospective study	16
Disability and health after replantation or revascularization in the upper extremity in a population in southern Sweden—a retrospective long time follow up	Retrospective study	326
Long-term outcome following upper extremity replantation after major traumatic amputation	Prospective study	16
Long-term functional, subjective and psychological results after single digit replantation	Prospective study	30
A four-year community hospital experience regarding procedures for the replantation and revascularization of fingers	Prospective study	58
Upper extremity replantation results in our series and review of replantation indications	Prospective study	14
Reconstructive surgery of the amputated ring finger	Retrospective study	9
Functional and subjective results of 20 thumb replantations	Prospective study	20
Long-term clinical results of 33 thumb replantations	Retrospective study	33
Functional and cosmetic outcome of single-digit ray amputation in hand	Prospective study	45

**Table 2:** Patient and injury characteristics.

Characteristics (*n* = 607)	
Age, y (mean)	38
Male	512
Female	95
Mechanism of injury (*n* = 518)	
Cut	407
Crush	80
Avulsion	26
Self-inflicted	5
Level of injury (*n* = 606)	
Arm	14
Elbow and forearm	31
Wrist	27
Metacarpal	29
Fingers	361
Thumb	144

**Table 3:** Functional scales and articles.

Scales	Number of Articles in Which They Appear
DASH (*n* = 160)	6
QuickDASH (*n* = 424)	3
CISS (*n* = 528)	7
Chen (*n* = 27)	2
MHQ (*n* = 46)	2
RAS (*n* = 54)	2
ROM (*n* = 87)	3
FIM (*n* = 16)	1
EQ-5D (*n* = 326)	1
SAS (*n* = 30)	1
BDI (*n* = 30)	1
SPTSS (*n* = 30)	1

## Data Availability

No new data were created or analyzed in this study. Data sharing is not applicable to this article.
